# Application of MPBT Assay for Multiplex Determination of Infectious Titers and for Selection of the Optimal Formulation for the Trivalent Novel Oral Poliovirus Vaccine

**DOI:** 10.3390/v16060961

**Published:** 2024-06-14

**Authors:** Hasmik Manukyan, Manjari Lal, Changcheng Zhu, Olga Singh, Tsai-Lien Lin, Erman Tritama, Konstantin Chumakov, Shwu-Maan Lee, Majid Laassri

**Affiliations:** 1 Center for Biologics Evaluation and Research, US Food and Drug Administration, 10903 New Hampshire Avenue, Silver Spring, MD 20993, USA; hasmik.manukyan@nih.gov (H.M.); olga.singh@fda.hhs.gov (O.S.); tsai-lien.lin@fda.hhs.gov (T.-L.L.);; 2 Center for Vaccine Innovation and Access, Program for Appropriate Technology in Health (PATH), Seattle, WA 98121, USA; mlal@path.org (M.L.); czhu@path.org (C.Z.); smlee@path.org (S.-M.L.); 3 Research and Development Division, PT Bio Farma, Bandung 40161, Indonesia; erman.tritama@biofarma.co.id

**Keywords:** nOPV, OPV, multiplex titration, poliovirus surveillance, clinical trials, thermal stability, vaccine formulation

## Abstract

Recently, a multiplex PCR-based titration (MPBT) assay was developed for simultaneous determination of infectious titers of all three Sabin strains of the oral poliovirus vaccine (OPV) to replace the conventional CCID_50_ assay, which is both time-consuming and laborious. The MPBT assay was shown to be reproducible, robust and sensitive. The conventional and MPBT assays showed similar results and sensitivity. The MPBT assay can be completed in two to three days, instead of ten days for the conventional assay. To prevent attenuated vaccine strains of poliovirus from reversion to virulence, a novel, genetically stable OPV (nOPV) was developed by modifying the genomes of conventional Sabin strains used in OPV. In this work, we evaluated the MPBT assay as a rapid screening tool to support trivalent nOPV (tnOPV) formulation development by simultaneous titration of the three nOPV strains to confirm stability as needed, for the selection of the lead tnOPV formulation candidate. We first assessed the ability of the MPBT assay to discriminate a 0.5 log_10_ titer difference by titrating the two tnOPV samples (undiluted and threefold-diluted) on the same plate. Once the assay was shown to be discriminating, we then tested different formulations of tnOPV drug products (DPs) that were subjected to different exposure times at 37 °C (untreated group and treated groups: 2 and 7 days at 37 °C), and to three freeze and thaw (FT) cycles. Final confirmation of the down selected formulation candidates was achieved by performing the conventional CCID_50_ assay, comparing the stability of untreated and treated groups and FT stability testing on the top three candidates. The results showed that the MPBT assay generates similar titers as the conventional assay. By testing two trivalent samples in the same plate, the assay can differentiate a 0.5 log_10_ difference between the titers of the tested nOPV samples. Also, the assay was able to detect the gradual degradation of nOPV viruses with different formulation compositions and under different time/temperature conditions and freeze/thaw cycles. We found that there were three tnOPV formulations which met the stability criteria of less than 0.5 log_10_ loss after 2 days’ exposure to 37 ℃ and after three FT cycles, maintaining the potency of all three serotypes in these formulations. The ability of the MPBT assay to titrate two tnOPV lots (six viruses) in the same plate makes it cheaper and gives it a higher throughput for rapid screening. The assay detected the gradual degradation of the tnOPV and was successful in the selection of optimal formulations for the tnOPV. The results demonstrated that the MPBT method can be used as a stability indicating assay to assess the thermal stability of the nOPV. It can be used for rapid virus titer determination during the vaccine manufacturing process, and in clinical trials. The MPBT assay can be automated and applied for other viruses, including those with no cytopathic effect.

## 1. Introduction

Polioviruses are members of the Enterovirus C species within the Picornaviridae family, which includes a diverse group of small viruses with single-stranded, positive RNA genomes [1,2]. There are three serotypes of poliovirus that are highly transmissible but rarely (less than 1% of infections) attack the central nervous system (CNS) and result in acute flaccid paralysis. There are two prophylactic vaccines: trivalent inactivated poliovirus vaccine (IPV) and trivalent live attenuated oral poliovirus vaccine (tOPV). Following introduction of these two vaccines, the cases of poliomyelitis dropped drastically worldwide.

The World Health Organization’s (WHO) Global Polio Eradication Initiative (GPEI) has made significant progress toward eradicating poliomyelitis. Wild poliovirus types 2 and 3 were declared eradicated in 2015 and 2019, respectively [3,4], and wild type-1 poliovirus was eliminated worldwide except for endemic regions in Afghanistan and Pakistan. However, Sabin strains used in OPV are genetically unstable and can revert to virulence and cause paralysis. The OPV vaccine recipients excrete revertant polioviruses [5] which can convert to pathogenic circulating vaccine-derived polioviruses (cVDPVs), causing outbreaks of paralytic disease [6]. Recently, a paralytic case caused by cVDPV2 was reported in an unvaccinated individual in Rockland County, New York [7].

Recently, two new genetically modified Sabin 2 strains were developed, resulting in two novel OPV2 (nOPV2) candidates [8,9]. Both candidates have domain V genetically stabilized by replacement of all U–G nucleotide pairs with C–G or U–A pairs [10]. nOPV2 candidate 1 (nOPV2-c1) was further stabilized by relocation of the recombination element (Cre) into the 5′ untranslated region and by insertion of the Asp_53_Asn and Lys_38_Arg mutations into the 3D polymerase to enhance fidelity and reduce recombination events, respectively [10,11]. In November 2020, the WHO issued an Emergency Use Listing (EUL) recommendation for nOPV2-c1 use during outbreak response. In December 2023, WHO pre-qualification (i.e., making sure that there is a sufficient supply of safe and effective vaccine for immunization programs) was granted to nOPV2-c1. For simplicity, candidate 1 is eliminated from the description of nOPV2 for the remainder of this manuscript. About 1 billion doses of nOPV2 have been used in 35 countries, with an excellent safety record [12,13]. Recently, similar genetically stable strains of serotypes 1 and 3 were developed using the nOPV2 genome as backbone; the capsid protein precursor P1 of nOPV2 was replaced with P1 of type 1 and 3 polioviruses to produce nOPV1 and nOPV3, respectively [14]. This opens the possibility of creating trivalent nOPV (tnOPV) composed of nOPV1, nOPV2 and nOPV3. Quantification of an infectious virus is a critically important task for the selection of the optimal formulation of trivalent vaccine and studying its stability. Traditionally, viruses are quantified either by plaque assay or by 50% cell culture infectious dose (CCID_50_) assay. Plaque assays require tedious visual counting of plaques which vary in size, complicating automated reading. The CCID_50_ assay requires monitoring of cytopathic effects (CPE), often over a long period of time [15,16,17]. In addition, the presence of several viruses in a mixture further complicates the task.

To overcome these limitations, we have previously developed a multiplex PCR-based titration (MPBT) assay [18] which simplified rapid titrations of OPV strains; where serial dilutions of viruses mixed with HEp-2C cells in 96-well plates are followed by two days of incubation, and cell lysates are then used for detection of the virus replication using multiplex quantification by qmosRT-PCR [19]. Endpoints obtained using the MPBT assay were similar to those obtained using the conventional CCID_50_ assay. The MPBT method is a simple, rapid, robust, reproducible, sensitive and suitable assay for multiplex titration of viruses.

In this work, we have explored the use of the MPBT assay for simultaneous titration of all three nOPV viruses and the ability of the MPBT assay to discriminate a 0.5 log_10_ titer difference by titrating the two trivalent nOPV (heat treated and untreated) side-by-side in the same plate. Using this approach, we conducted screening of twelve tnOPV formulations to select the most thermal stable formulation for the tnOPV using a tiered approach, where the formulation candidates which showed the lowest loss of potency after incubation for 2 and 7 days, respectively, at 37 °C were advanced for evaluation of freeze–thaw stability through a treatment of multiple freeze–thaw cycles.

Our results show that the MPBT assay can titrate two tnOPV formulation samples (six viruses) for comparison in the same plate, enabling a high throughput for rapid selection of the lead tnOPV formulation. This assay format can be used for rapid assessment of the thermal stability of nOPV, as well as for rapid virus titer determination during the vaccine manufacturing process and in clinical trials.

## 2. Materials and Methods

### 2.1. nOPV Samples and HEp-2C Cells

Monovalent bulks of nOPV1, nOPV2 and nOPV3 were provided by PT Bio Farma (Bandung, Indonesia). The formulated tnOPV Dug products (DP) that are listed in Table 1 and the heat-stressed samples were provided by PATH. These samples were used for titer determination.

HEp-2C cells (ATCC^®^ CCL-23^™^) were grown in 150 cm^2^ flasks at 37 °C ± 2 °C in Dulbecco’s modified eagle medium (DMEM; Gibco) supplemented with 5% fetal calf serum (FCS; Gibco) and penicillin-streptomycin (at final concentrations of 100 U/mL and 100 µg/mL, respectively; Gibco). Cell counting was performed on stained cell samples with Trypan Blue (Invitrogen, Waltham, MA, USA) and viable cells counted with a hemocytometer (Countess^TM^; Thermo Fisher, Waltham, MA, USA). HEp-2C cell numbers of 2 × 10^4^ and 4 × 10^4^ were used for the CCID_50_ and MPBT assays, respectively.

### 2.2. Virus Titration by CCID_50_ Assay

nOPV viruses were titrated by the cell culture infectious dose (CCID_50_) assay on HEp-2C cells. The assay was performed by adding serial threefold or twofold dilutions of viral samples in 100 µL of DMEM supplemented with 2% FCS in 96-well plates. 100 µL aliquots of cell suspension containing 2 × 10^4^ HEp-2C cells in DMEM with 2% FCS were added to each well of diluted virus in replicate wells of 96-well plates. Virus-infected plates were incubated for 10 days at 33 °C in a 5% CO_2_ humidified air atmosphere; wells were periodically examined for cells’ cytopathogenic effect (CPE) for 10 days. Wells showing CPE were counted and virus titers calculated using the Spearman–Karber formula [20]. For tnOPV assay by CCID_50_, type-specific polyclonal antisera were used to measure 1 type of virus at a time after neutralizing the other 2 types of viruses, as described previously [21,22].

### 2.3. Multiplex PCR-Based Titration (MPBT) Assay

The MPBT assay was performed as described previously [18] with some changes. Serial threefold or twofold dilutions of viral samples were prepared and mixed with 4 × 10^4^ HEp-2C cells in 96-well plates as described above for the CCID_50_ assay, with the following exceptions: plates were incubated for only 42 h, after which the medium was discarded, the cells were washed with 150–200 µL 1× PBS (prepared from 10× Phosphate Buffered Saline), then 50 µL of lysis solution (DMEM with 0.9% Triton X-100) was added to each well. The plates were then sealed with foil (Thermo Fisher) and stored at −80 °C prior to analyzing them with the updated quantitative multiplex one-step RT-PCR (qmosRT-PCR) assay [23]. The most important change to the MPBT assay was the use of the updated qmosRT-PCR assay as readout method; this updated assay uses the QuantiNova Multiplex RT-PCR Kit (QIAGEN, Valencia, CA, USA) instead of the QuantiFast Multiplex RT-PCR Kit (QIAGEN, Valencia, CA, USA) that is out of production, and the reverse primers for types 1 and 3 were redesigned to increase the sensitivity for the detection of poliovirus type 1 and 3 [23].

The plates with lysed cells were thawed for 30 min at room temperature and briefly centrifuged to collect the lysate at the bottom of the wells. Dilutions of 1:10 of the cell lysates were then prepared and subjected to qmosRT-PCR analysis [23]. The samples and the qmosRT-PCR reactions were aseptically prepared in a class II biosafety cabinet (The Baker company, Sanford, ME, USA).

Samples that have Cts less than or equal to 35 are considered positive and samples with Cts higher than 35 are considered negative. The PCR reaction was considered invalid if positive control was negative and/or negative control was positive. Each well of the plate was scored positive or negative for the presence of the virus activity (virus genome replication) and used for titer calculation for each nOPV serotype according to the following Spearman–Karber formula [20]: log_10_ CCID_50_ = −(L − d(S − 0.5)) where, L is the log_10_ of lowest dilution in test, d is the difference between log10 dilutions and S is the sum of proportions of positive tests (i.e., cell cultures that were shown to be positive for virus genome replication by qmosRT-PCR method).

A simplex PCR-based titration (SPBT) assay was used in some comparative experiments to analyze monovalent samples. The SPBT uses the same condition and reagents of the MPBT assay as described above, with the only exception that it only uses the set of primers and probe that is specific for the targeted poliovirus type.

### 2.4. Formulation Preparation

A total of twelve liquid tnOPV formulations (referred to as drug products in the text) were developed by combining the bulk nOPV viruses (type 1, 2 and 3) provided by the vaccine manufacturer, PT Bio Farma (Bandung, Indonesia) with various stabilizing excipients such as sugars, amino acids, magnesium salts and polyethylene glycol in varying ratios at PATH lab. The composition of the developed formulations is shown in Table 1. The excipients were selected based on the published literature, for their ability to stabilize the poliovirus, especially from temperature-induced degradation. Formulations were prepared at 10–20 mL scale in glass vials. For each formulation, the respective excipient mix was prepared at twofold (2×) or fourfold (4×) of the final concentration in Basal Medium Eagle, with Earle’s salt, with L-glutamine, without Sodium bicarbonate (BME, Gibco), mixed with the calculated amount of bulk virus, QS with BME and pH adjusted to target of 6.5 using Sodium bicarbonate or Acetic acid. Once prepared, each liquid tnOPV formulation was aliquoted into 1 mL per cryovial and split into 3 treatment groups: (A) Treated for 2 days at 37 °C (T2d), (B) Treated for 7 days at 37 °C (T7d), (C) Untreated (UT or T0). The UT group aliquots were frozen at –80 °C immediately after preparation. The T2d and T7d groups were frozen at –80 °C after completion of the temperature exposure conditions. All 3 groups were shipped together frozen to the FDA lab for MPBT assay testing.

### 2.5. Freeze/Thaw Samples Preparation

One milliliter tubes of the respective formulations 4, 7, 9, 10, 11 and 13 (Table 1) were subjected to multiple freeze–thaw cycles. Briefly, we removed the tube from freeze temperature (≤–80 °C), thawed it at 16–25 °C (room temperature) for 4 h. After the first thaw, we placed the sample tube back at ≤–80 °C, upright position for 20 h, then we performed second thaw at room temperature, for 4 h, after the second thaw we placed the sample tube back at ≤–80 °C (upright position) for 20 h, and in the end, we preformed the third thaw at room temperature, for 4 h. After completion of the third thaw cycle, we tested the sample on the same day using MPBT assay as described above.

## 3. Results

### 3.1. Evaluation and Optimization of MPBT Assay for Titration of Trivalent nOPV (tnOPV)

To demonstrate the performance of the MPBT assay for titer determination of tnOPV viruses, nOPV1, 2 and 3 monovalent bulks were subjected to a conventional CCID_50_ assay and single-plex PCR titration (SPBT) and after combining the three serotypes, subjected to the MPBT assay using the entire 96-well plate, 10^−5^ as starting dilution, twofold serial dilution and eight repeats on the plates (two runs each). The obtained results (Table 2, top portion) showed that the determined titers between all three assays and for all three types of tnOPV are within the acceptable variation of ±0.5 log_10_ [24], indicating that the MPBT and CCID_50_ assays generate similar results and the MPBT assay can be used for simultaneous titration of the three types of the tnOPV.

The MPBT assay is going through this evaluation to be used for titration of the three viruses of the tnOPV drug product (DP), that have known titers of about 7.3 log_10_CCID_50_/mL for type 1, 6.6 log_10_CCID_50_/mL for type 2 and 7.3 log_10_CCID_50_/mL for type 3. To assess the possibility to run two tnOPV DP samples in the same plate using the MPBT assay, nOPV 1, 2 and 3 monovalent bulks were subjected to two runs of the CCID_50_ assay, SPBT assay and MPBT assay (for trivalent nOPV) using half plate, 10^−5^ (for lot 2) and 10^−6^ (for lot 1) as starting dilution, seven threefold serial dilutions and six repeats. The generated results (Table 2) from the three assays are within the acceptance variation of ±0.5 log_10_ [24] when the methods (CCID_50_, SPBT and MPBT) were used under the same conditions (starting dilution, plate dilutions and repeats). Also, the results of the analysis of the same tnOPV lot (lot 1) using half plate and whole plate (Table 2, portions 1 and 2) for each nOPV type met the ±0.5 log10 acceptance criterion within each of the three assays. The results (Table 2) demonstrate that the CCID_50_ and MPBT assays generate similar results for all three nOPV serotypes. Therefore, the MPBT assay can be used for titration of the tnOPV in half plate, which reduced considerably the price of the assay (i.e., simultaneous titration of six viruses in the same plate).

### 3.2. Evaluation of the MPBT Assay’s Ability to Differentiate 0.5 Log_10_ Difference between Titers Using Half Plate

To evaluate the ability of the MPBT assay to differentiate 0.5 log_10_ titer, two operators titrated a tnOPV sample 1 and its threefold dilution (threefold dilution reduced the titer to about 0.5 log_10_) with two runs of the MPBT assay on days 1 and 2 using half plate, 10^−6^ starting dilution and seven threefold serial dilutions. Both operators’ results (Table 3) showed that the assay can differentiate 0.5 log_10_ titer. In addition, operator 1 subjected another tnOPV sample 2 and its threefold dilution to two runs of the MPBT assay under the same condition used for sample 1 above. The obtained results (Table 3) on days 1 (operator 1) and 2 (operator 2) for sample 1 showed that the assay generated reproducible results (within the acceptance specification of ±0.5 log_10_ variation [24]). Also, the results of the analysis of samples 1 and 2 demonstrated that the assay is able to discriminate a 0.5 log_10_ titer difference. The titer differences between non-diluted and threefold-diluted samples are highly significant (*p*-values < 0.001) for all three serotypes.

### 3.3. MPBT Assay Evaluation for Titer Determination of Untreated and Treated 35% Sucrose Formulated tnOPV Drug Product

We evaluated the ability of the MPBT assay to determine the titer of the formulated tnOPV drug product (DP) by analyzing 35% sucrose formulated DP in 3 different days with three runs per day using full plate: 10^−5^ as starting dilution, 11 twofold serial dilution and 8 well-repeats. The results (Table 4) showed that the generated titers were consistent from day to day and run to run for the three nOPV types (the results were within the acceptance variation of ±0.5 log_10_ [24]). Also, the intermediate precision (%CV) estimated from this dataset was 36.76%, 38.39% and 26.49% for serotypes 1, 2 and 3, respectively, which was considered good for this type of assay. The MPBT assay can be used for titration of formulated tnOPV drug products.

**Figure 1:**
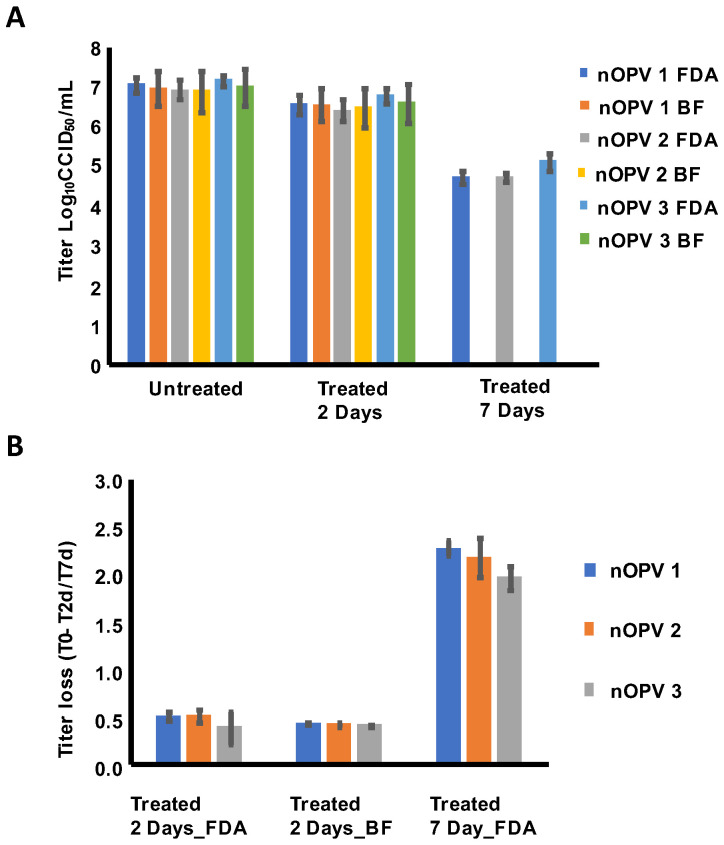
Titer determination of the 35% sucrose formulated tnOPV DP that was untreated (T0), treated for 2 days at 37 °C (T2d) and 7 days at 37 °C (T7d). (**A**) Titers determined in US Food and Drug Administration (FDA) laboratory using MPBT assay and at BioFarma (BF) using the CCID_50_ assay. (**B**) Titer loss determined between titers of T0 and T2d/T7d samples generated at FDA and BF.

To detect the difference in titer between the 35% sucrose formulated tnOPV DP that was untreated (T0) and treated for 2 days at 37 °C (T2d), at the US Food and Drug Administration (FDA) laboratory, we analyzed the untreated and treated samples by MPBT assay in the same plate, and the same 35% sucrose formulation was analyzed at BioFarma (BF) using the CCID_50_ assay; both assays were run in 3 days (three runs per day) resulting in nine titer values for each nOPV type. The results (Figure 1A) showed that the average titers obtained by FDA and BF for T0 and T2d samples are similar as they are within the acceptance specification of ±0.5 log_10_ variation [23], and the titer differences between T0 and T2d samples are similar (less than twofold difference) between the two institutions (Figure 1B) with ratio T0-T2d(FDA)/T0-2d(BF) being 1.18 for type 1, 1.21 for type 2 and 0.94 for type 3. These results showed that 37 °C reduced the titer of the 35% sucrose formulated tnOPV DP for about 0.5 log_10_ after 2 days. These results also confirmed that the MPBT and CCID_50_ assays generate similar results.

To detect the difference in titer between the 35% sucrose formulated tnOPV DP that was untreated (T0) and treated for 7 days at 37 °C (T7d) at the FDA laboratory, we analyzed the untreated and treated samples using the MPBT assay in the same plate. The assay was run on 2 different days (three runs per day), resulting in six titer values for each nOPV type. The results (Figure 1A,B) showed that the titers dropped drastically after 7 days of treatment. These results (Figure 1B) showed that treatment at 37 °C reduced the titer of the 35% sucrose formulated tnOPV DP by about 2 log_10_ after 7 days.

### 3.4. MPBT Assay Analysis of Untreated, Treated and Freeze–Thawed tnOPV Drug Products Formulated with Different Excipients

**Figure 2:**
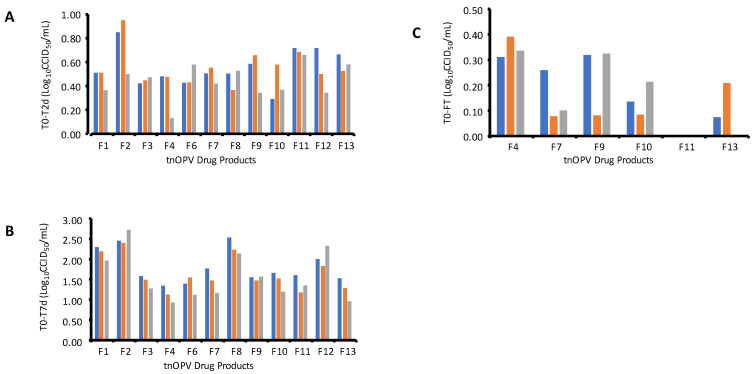
Titer losses between the untreated and treated of all tnOPV drug product formulations (Table 1). (**A**) Titer losses between untreated (T0) and treated 2 days at 37 °C (T2d). (**B**) Titer losses between untreated (T0) and treated 7 days at 37 °C (T7d). (**C**) Titer losses between untreated (T0) and freeze–thaw treated (FT) of the selected tnOPV Drug Products (i.e., F4, F7, F9, F10, F11 and F13 samples). 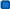
—nOPV1, 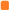
—nOPV2, 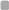
—nOPV3.

To select the optimal formulation that can improve the thermal stability of the tnOPV, we analyzed all tnOPV drug product samples with different formulation compositions listed in Table 1. The samples were subjected to the thermal treatments; the untreated (T0) and thermal treated at 37 °C for 2 days (T2d) and for 7 days (T7d) samples were run three times by the MPBT assay as described above for the analysis of the 35% sucrose formulated tnOPV samples. The titer results for T0/T2d and T0/T7d are presented in supplementary Tables S1 and S2, respectively, and the summary of the mean titer differences (titer loss) T0-T2d and T0-T7d are presented in Figure 2A and Figure 2B, respectively. Most formulations resulted in a mean titer difference T0-T2d of about 0.5 log_10,_ except for the nOPV types 1 and 2 of the formulation 2 that have T0-T2d close to 1.0 log_10_ (Figure 2A). Also, we observed that most formulations resulted in a difference T0-T7d of about 1.5 log_10_ or less, except for formulations 1, 2, 8 and 12 which resulted in T0-T7d of about 2 log_10_ or higher (Figure 2B). Between the formulations that showed good thermal stability at 37 °C for seven days, the formulations 4, 7, 9, 10, 11 and 13 were selected for further treatment with three cycles of freeze–thaw (FT) as described above. The titer results are presented in supplementary Table S3 and Figure 2C. The temperature stress and freeze–thaw data results ranked formulations 7, 10 and 11 as the promising formulation candidates.

The selection criteria for formulation down selection were based on a combination of factors including (a) thermo stability, (b) freeze–thaw stability, (c) palatability and (d) prior use of excipients in vaccines. Although several formulations met the (a) and (b) criteria, taste is also an important consideration since this is an oral vaccine, primarily targeting infants. Formulation F3 did meet the stability criteria, however magnesium formulation alone would likely have a strong metallic taste (bitter), potentially impacting acceptability in the target patient population [25,26]. Similarly, Formulation F13 (sucrose with arginine) met most of the selection criteria, however, F11 (sucrose with histidine) formulation was superior because the effective buffering range of histidine (pKa of imidazole) is from 5.12–7.2, which is appropriate for maintaining the stability of the nOPV at pH 6.5–7.2. Therefore, F13 (pKa guanidinium group is 12.5) was not selected with the top running candidates.

**Figure 3:**
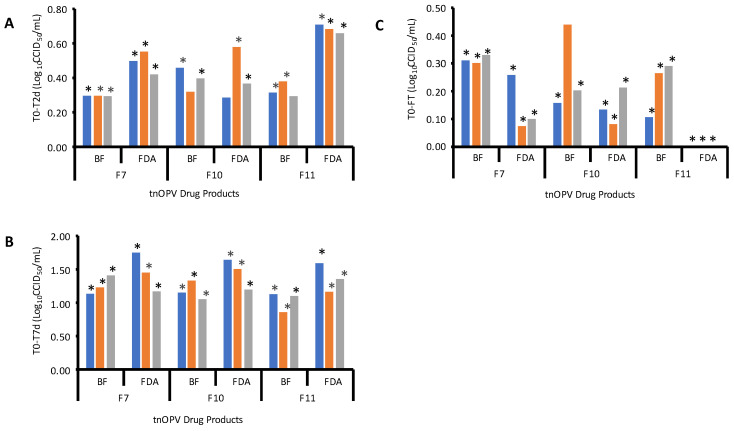
Titer losses (generated by FDA using MPBT assay and by BioFarma using CCID_50_ assay) between the untreated and treated samples for the 3 lead formulations. (**A**) Titer losses between untreated (T0) and treated 2 days at 37 °C (T2d). (**B**) Titer losses between untreated (T0) and treated 7 days at 37 °C (T7d). (**C**) Titer losses between untreated (T0) and freeze–thaw treated (FT) of the selected tnOPV Drug Products. 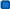
—nOPV1, 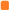
—nOPV2, 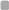
—nOPV3. * For (**A**,**B**), *p*-value < 0.05 for regular significance test indicates that significant titer loss from T0 to T2d (**A**) or T7d (**B**) is detected. For comparison between T0 and FT3 (**C**) within each institution, where the goal is to confirm the comparability between institutions, *p*-value < 0.05 for equivalence test or 90% CI for difference within ±0.5 log_10_ equivalence margin is required to demonstrate equivalence.

The confirmation of the three down selected formulations (7, 10 and 11) was performed at the BioFarma facility using their established CCID_50_ assay for types 1, 2 and 3. The results from this testing are presented in Figure 3. Figure 3A shows the log_10_ loss of titer following the thermal stress of 2 days’ incubation at 37 °C, Figure 3B is the log_10_ loss of titer after 7 days of thermal stress at 37 °C, and Figure 3C shows log_10_ loss of titer after subjecting the formulations to three freeze–thaw cycles. The stability results on the three down selected tnOPV formulation candidates from the two institutions (results generated by the MPBT assay at the FDA laboratory and by the CCID_50_ assay at BioFarma) showed that *p*-values < 0.05 for differences of T0-T2d (Figure 3A), T0-T7d (Figure 3B) were observed in all data; with a few exceptions where *p*-value was >0.05 likely due to the small number of data (titers) generated in these experiments, suggesting that both assays are stability indicating. The *p*-value for equivalence testing (similarity demonstrated if *p*-value is <0.05 for the equivalence hypothesis, i.e., the 90% CI is contained in the ±0.5 log_10_ margin) showed that in each institution, the FT3 titers were similar to the T0 titers. For between-assay comparison (FDA MPBT versus BioFarma CCID_50_), equivalence was demonstrated with respect to the difference in T0-FT3 (Figure 3C), suggesting that the two test methods are equivalent. The equivalence between the two methods was not consistently demonstrated across formulations and serotypes for T0-T2d and T0-T7d as for T0-FT3 (most likely due to insufficient power), although a general trend was displayed, and all estimated between-assay differences were within a ±0.5 log_10_ interval.

The final selection of the lead tnOPV formulation will be determined based on the outcome of the stability testing of the scale-up batches (ongoing study).

## 4. Discussion

The plaque and CCID_50_ are the conventional assays used for quantification of viruses. The viral plaque assay is one of the most commonly used method in virology to select viral clones or to determine virus titers as plaque formation units per milliliter [27,28,29,30,31]. However, viral CCID_50_ and plaque assays are time-consuming, requiring four to twelve days, depending on the virus type, and ten days for nOPV viruses [15,16,17,32]. Furthermore, plaque assays work only for viruses capable of lysing the cells. Similarly, CCID_50_ assays are used to quantitate viruses that induce CPE in cell cultures [15,16,17,32]. But not all viruses are able to induce CPE in the cells they infect. Titration results of both the plaque and CCID_50_ assays are slightly variable, with more than a 10% error rate [33]. Both conventional assays are difficult to automate, and neither is suitable at the large scales needed for clinical and environmental samples’ analysis. Without further modification, neither assay works easily with samples containing more than one virus, such as trivalent OPV/nOPV.

To overcome these limitations, we developed the MPBT assay [18] as a straightforward and fast alternative to conventional CCID_50_ assays to detect, identify and titrate either individual or multiple serotypes of OPV. The MPBT method is suitable for identifying and quantifying polioviruses in large numbers of specimens collected in clinical trials of poliovirus vaccines (nOPV/OPV). Titration of trivalent vaccine with the MPBT assay is superior to the conventional assay in term of time and labor, and it can titrate six viruses in the same plate “2 trivalent vaccine samples” in 2 days, which makes it costless. By contrast, the conventional assay requires the introduction of a neutralization step before titration, titrates only one virus per run and takes 10 days to get the results. The MPBT assay is easy and safe to execute: it uses qmosRT-PCR assay as readout method (instead of CPE which is used in the conventional assay), and is performed in a biosafety cabinet that provides personnel, environmental and product protection.

The MPBT is a multiplex assay: it has the potential to be used for quality control during manufacture of poliovirus vaccines, identification of vaccine poliovirus serotypes (potentially replacing the current sero-neutralization identity test), monitoring inactivation kinetics and measuring the potency. This assay is appropriate for automation, simplifying high-throughput applications, improving consistency of titrations and saving time, cost and labor.

Recently, a novel type 2 oral poliovirus vaccine candidate 1 (nOPV2), produced by genetic modification of the type 2 Sabin vaccine virus genome [9], was shown to be safe and well-tolerated, have noninferior immunogenicity and have superior genetic stability compared to Sabin monovalent type 2 [34]. This vaccine was pre-qualified by the WHO in December 2023 [12]. nOPV 1 and 3 were produced by replacing the P1 genome region (entire capsid proteins) of the nOPV2 by the P1 region of polioviruses types 1 and 3, respectively [14]. The trivalent nOPV (tnOPV) is composed of nOPV2, nOPV1 and nOPV3 viruses. Under this work, the formulation efforts focused on developing novel tnOPV formulations that stabilize all three serotypes for at least 2 days at 37 °C, as required by Vaccine Vial Monitor 2 (VVM 2). The acceptable criteria for a vaccine with a VVM label are determined by the specific vaccine’s stability profile. For polio vaccines, to meet the VVM2 criteria, the stability of the live attenuated virus must be demonstrated to be <0.5 log_10_ loss at 37 °C for 2 days. This criteria of <0.5 log_10_ loss at 37 °C for 2 days was our minimal down selection criteria. Traditional Sabin OPV formulations are formulated with 35% sucrose as a stabilizer, where significant bulk volume is occupied by sucrose. This conventional formulation method does not achieve high virus concentrations in the final drug product. Therefore, novel formulations were developed to allow higher concentrations of nOPV serotypes to be accommodated in the trivalent nOPV formulation. For tnOPV, the new formulation allowed more tnOPV bulk virus to be accommodated in the final formulation (DP), while meeting the VVM2 requirements for the vaccine.

In summary, our approaches were: (1) To develop tnOPV vaccine without increasing the 0.1mL dose volume used for bOPV [35] or tOPV (in 2016, the use of tOPV was discontinued and replaced with bOPV and at least one dose of IPV [36]) formulation, (2) To formulate tnOPV at a higher virus concentration than tOPV by cutting sucrose concentration in the formulation or replacing/combining with another excipient, (3) Adding an excipient to provide buffering capacity, (4) Adding stabilizing excipients to meet VVM2 criteria.

In this work, we first demonstrated the possibility of using the MPBT assay for simultaneous titer determination of the three tnOPV viruses by comparing the conventional CCID_50_ and MPBT assays. The results of both assays were consistent based on the titer for each serotype; we then optimized the MPBT assay to analyze two tnOPV samples (six viruses) in the same plate by selecting the optimal starting dilution (10^−4^–10^−5^), and seven threefold serial dilutions. The assay was shown to be reproducible and sensitive, capable of detecting a 0.5 log_10_ titer difference for each serotype.

In this work, and previously [18], we observed a good correlation between the CCID_50_ and MPBT assays. There are different factors contributing to this correlation: the first factor is the design of the MPBT assay that allows us to measure the virus activity based on the detection of the virus genome replication inside the cells [18]; and the second important factor is the use of the optimized Qiagen one-step RT-PCR kit, which efficiently detects the virus genome as it is known that different one-step RT-PCR kits have different levels of amplification efficiency for the detection of viruses [23,37].

Next, we explored the suitability of the MPBT assay as a simple, rapid and high-resolution analytical tool for evaluating the thermal stability of different tnOPV drug product (DP) formulations (Table 1), with sensitivity to detect a 0.5 log_10_ titer difference for each serotype in the tnOPV formulations. The results showed that the logarithmic mean differences between T0 and T2d, and between T0 and T7d vary from 0.13 to 0.95 and from 0.92 to 2.72 (Supplementary Tables S1 and S2, Figure 2A,B), respectively, depending on the formulation and nOPV type. Based on these results, formulations 4, 7, 9, 10, 11 and 13 were selected for further analysis with the MPBT assay following three freeze–thaw (FT) cycles at room temperature, as described in the Materials and Methods section above. After the combined evaluation of the results from temperature stability and FT stability testing, three formulations (7, 10 and 11) were downselected based on the MPBT assay and further confirmed using the CCID_50_ assay performed at BioFarma, where comparable log_10_ losses in titer were observed for the three downselected formulation candidates.

Histidine containing formulations 7 and 11 offered the additional benefit of buffering capacity, with pKa being 6.0 for histidine. The original sucrose formulation (35% sucrose) is poorly buffered; the limited buffering capacity came from pH adjustment where sodium bicarbonate and acetic acid were used. Over long-term storage, the pH gradually went up as a result of losing CO_2_ and maintaining pH at 6.5–7.2 over the long shelf life became a challenge. The new histidine-containing formulation would improve pH stability during storage. The arginine-containing formulation would not have the same benefit because the pka of arginine was 8.99.

In this communication, we showed that the MPBT is a simple, rapid and accurate assay for detection, identification and titration of either individual or combined serotypes of tnOPV polioviruses. The MPBT method can run two tnOPV samples (six viruses) in the same plate, which increases the throughput and decreases the cost of the assay. The assay was successfully applied for rapid screening and down selection of the optimal thermostable formulations for the tnOPV, based on 37 ℃ and FT stability. These results suggest that the MPBT assay has the potential to be used as a quality control tool during the manufacture of poliovirus vaccines.

## Figures and Tables

**Table 1:** Proposed tnOPV drug product formulations composition.

Formulation	Composition (W/V) %	tnOPV Dug Product Titer
F1	35% Sucrose *	Type 1 Titer: log_10_ 7.3 CCID_50_/mL Type 2 Titer: log_10_ 6.6 CCID_50_/mL Type 3 Titer: log_10_ 7.3 CCID_50_/mL
F2	28% Sucrose	
F3	1M MgCl_2_ 6H_2_O	
F4	1M MgCl_2_ 6H_2_O + 0.05% PS80	
F6	28% Sucrose + 1M MgCl_2_.6H_2_O	
F7	28% Sucrose + 0.5% Histidine	
F8	28% Sucrose + 0.5% PEG8000	
F9	28% Sucrose + 0.5% Arginine	
F10	14% Sucrose + 1M MgCl_2_.6H_2_O	
F11	14% Sucrose + 0.5% Histidine	
F12	14% Sucrose + 0.5% PEG8000	
F13	14% Sucrose + 0.5% Arginine	

Note: * First formulation subjected to MPBT assay. DP; Drug product.

**Table 2:** Evaluation and optimization of MPBT assay for titration of trivalent nOPV using whole and half 96-well plate.

nOPV Type (Lot)	CCID_50_ Assay			SPBT Assay			MPBT Assay			CCID_50_ Assay	
	Run 1	Run 2	Mean ± SD	Run 1	Run2	Mean ± SD	Run 1	Run 2	Mean ± SD	±0.5 log_10_ Range	
11 twofold serial dilutions, 8 repeats, whole plate, and starting dilution is 10^−5^										LL	UL
1 (1)	8.03	8.07	8.05 ± 0.03	8.30	8.03	8.16 ± 0.19	7.96	8.30	8.13 ± 0.24	7.55	8.55
2 (1)	7.92	7.99	7.96 ± 0.05	8.22	8.07	8.14 ± 0.11	8.41	8.22	8.31 ± 0.13	7.46	8.46
3 (1)	7.58	7.66	7.62 ± 0.05	7.66	7.77	7.71 ± 0.08	7.77	7.54	7.66 ± 0.16	7.12	8.12
7 threefold serial dilutions, 6 repeats, half plate, starting dilution is 10^−6^											
1 (1)	7.72	7.80	7.76 ± 0.06	7.87	8.19	8.03 ± 0.22	8.19	7.72	7.95 ± 0.34	7.26	8.26
2 (1)	7.95	7.87	7.91 ± 0.06	7.80	8.27	8.03 ± 0.34	8.11	7.87	7.99 ± 0.17	7.41	8.41
3 (1)	7.72	8.11	7.91 ± 0.28	7.95	7.72	7.83 ± 0.17	7.72	7.72	7.72 ± 0	7.41	8.41
7 threefold serial dilutions, 6 repeats, half plate, and starting dilution is 10^−5^											
1 (2)	7.67	7.59	7.63 ± 0.06	7.67	7.75	7.71 ± 0.06	7.51	7.67	7.59 ± 0.11	7.13	8.13
2 (2)	6.95	7.35	7.15 ± 0.28	7.11	6.95	7.03 ± 0.11	7.43	7.67	7.55 ± 0.17	6.65	7.65
3 (2)	8.47	8.15	8.31 ± 0.22	7.67	7.51	7.59 ± 0.11	7.75	7.83	7.79 ± 0.06	7.81	8.81

Note: SD; Standard deviation of log_10_ values. The titer unit is log_10_CCID_50_/mL. LL; Lower limit of the range. UL; Upper limit of the range.

**Table 3:** Evaluation of the ability of the MPBT assay to differentiate 0.5 Log_10_ of titer using half plate, seven threefold dilutions, and six repeats.

nOPV Type	Sample Titer	MPBT Assay, Log_10_ titers						Log_10_ (Difference of Mean) Titers
		Non-Diluted Sample			Threefold Dilution of the Sample			
		Run 1	Run 2	Mean	Run 1	Run 2	Mean	
tnOPV sample 1 run by operator 1 on day 1								
1	7.83	7.99	7.83	7.91	7.19	7.43	7.31	0.60
2	7.85	8.15	7.91	8.03	7.51	7.51	7.51	0.52
3	7.69	7.83	8.07	7.95	7.35	7.51	7.43	0.52
tnOPV sample 1 run by operator 2 on day 2								
1	7.83	7.91	7.91	7.91	7.59	7.51	7.55	0.36
2	7.85	7.83	8.07	7.95	7.67	7.83	7.75	0.20
3	7.69	8.07	8.31	8.19	7.59	7.51	7.55	0.64
tnOPV sample 2 run by operator 1 on day 2								
1	7.59	8.07	7.83	7.95	7.19	7.03	7.11	0.83
2	7.55	7.51	7.43	7.47	7.11	7.03	7.07	0.40
3	7.79	7.99	7.75	7.87	7.59	7.19	7.39	0.48
	Comparison between non-diluted versus threefold-diluted							
nOPV type	Delta: log_10_ Observed Titer—Expected log_10_ Sample Titer				Difference between dilutions			
	Non-diluted		Threefold-diluted		Non-diluted—threefold-diluted			
	Mean	SD	Mean	SD	Mean Difference		*p*-value	
1	0.17	0.17	−0.43	0.15	0.60		0.0001	
2	0.07	0.16	−0.31	0.18	0.37		0.0006	
3	0.28	0.23	−0.27	0.18	0.55		0.0004	

Note: non-diluted and threefold-diluted samples were run in the same plate using 6 well-repeated per dilution. An ANOVA model with sample and dilution as the independent variables was fitted to the log10 titer data to assess the effect of dilution treatment. *p*-value is for testing a null hypothesis of no difference. A *p*-value of <0.05 indicates that a significant difference is detected.

**Table 4:** MPBT assay titration of 35% sucrose formulated tnOPV DP using 11 twofold serial dilutions, 8 well-repeats and the entire plate.

Day	nOPV Type	MPBT Assay (log_10_CCID_50_/mL)				Intermediate Precision per Type	
		Run 1	Run 2	Run 3	Mean ± SD	Mean * ± SD * (Type)	%CV * (Type)
1	1	7.58	7.32	7.39	7.43 ± 0.14	7.3 ± 0.15 (1)	36.76% (1)
	2	7.09	7.24	7.20	7.18 ± 0.08	7.14 ± 0.16 (2)	38.39% (2)
	3	7.39	7.17	7.51	7.35 ± 0.17	7.29 ± 0.11 (3)	26.49% (3)
2	1	7.35	7.24	7.35	7.32 ± 0.07		
	2	7.24	7.13	7.39	7.25 ± 0.13		
	3	7.32	7.28	7.35	7.32 ± 0.04		
3	1	7.02	7.17	7.28	7.15 ± 0.13		
	2	6.90	7.20	6.90	7.00 ± 0.17		
	3	7.17	7.20	7.24	7.2 ± 0.04		

Note: SD; Standard deviation. The expected titer of the tested tnOPV samples is of about 7.3, 6.6 and 7.3 log_10_CCID_50_/mL for type 1, 2 and 3, respectively. CV; Coefficient of variation. *; Mean, SD and %CV are calculated from all titer values of each type presented in this table.

## Data Availability

All relevant data are within the paper.

## Supplementary Materials

The following supporting information can be downloaded at: https://www.mdpi.com/article/10.3390/v16060961/s1, Table S1: MPBT assay titer determination of tnOPV drug products with different formulations; untreated (T0) and treated 2 days at 37 °C (T2d); Table S2: MPBT assay titer determination of the tnOPV drug products with different formulations; untreated (T0) and treated for 7 days at 37 °C (T7d); Table S3: MPBT assay titer determination of tnOPV drug products with different formulations ; untreated (T0) and treated with freeze -thaw cycles (FT).

## Abbreviations

FDA: Food and Drug Administration; BF: BioFarma; CPE: Cytopathic effect; MPBT: Multiplex PCR-Based Titration; SPBT: Simplex PCR-Based Titration tnOPV: trivalent novel Oral Poliovirus Vaccine; OPV: Oral Poliovirus Vaccine; IPV: Inactivated Poliovirus Vaccine; CCID50: Cell Culture Infectious Dose 50%; PFU: Plaque Formation Unite; RT-PCR: Reverse-Transcriptase Polymerase Chain Reaction; qmosRT-PCR: Quantitative Multiplex One-Step Reverse-Transcriptase Polymerase Chain Reaction; WHO: World Health Organization; GPEI: Global Polio Eradication Initiative; DP: Drug Product; VVM2: Vaccine Vial Monitor 2; FT: Freeze–Thaw.
